# Molecular Breeding of Rice Restorer Lines and Hybrids for Brown Planthopper (BPH) Resistance Using the *Bph14* and *Bph15* Genes

**DOI:** 10.1186/s12284-016-0126-1

**Published:** 2016-10-04

**Authors:** Hongbo Wang, Shengtuo Ye, Tongmin Mou

**Affiliations:** National Key Laboratory of Crop Genetic Improvement and National Center of Plant Gene Research (Wuhan), Huazhong Agricultural University, Wuhan, 430070 China

**Keywords:** Rice, Brown Planthopper (BPH), *Bph14* and *Bph15*, MAS, Hybrid Rice

## Abstract

**Background:**

The development of hybrid rice is a practical approach for increasing rice production. However, the brown planthopper (BPH), *Nilaparvata lugens* Stål, causes severe yield loss of rice (*Oryza sativa* L.) and can threaten food security. Therefore, breeding hybrid rice resistant to BPH is the most effective and economical strategy to maintain high and stable production. Fortunately, numerous BPH resistance genes have been identified, and abundant linkage markers are available for molecular marker-assisted selection (MAS) in breeding programs. Hence, we pyramided two BPH resistance genes, *Bph14* and *Bph15*, into a susceptive CMS restorer line Huahui938 and its derived hybrids using MAS to improve the BPH resistance of hybrid rice.

**Results:**

Three near-isogenic lines (NILs) with pyramided *Bph14* and *Bph15* were obtained by molecular marker-assisted backcross (MAB) and phenotypic selection. The genomic components of these NILs were detected using the whole-genome SNP (Single nucleotide polymorphism) array, RICE6K, suggesting that the recurrent parent genome (RPG) recovery of the NILs was 87.88, 87.70 and 86.62 %, respectively. BPH bioassays showed that the improved NILs and their derived hybrids carrying homozygous *Bph14* and *Bph15* were resistant to BPH. However, the hybrids with heterozygous *Bph14* and *Bph15* remained susceptible to BPH. The developed NILs showed no significant differences in major agronomic traits and rice qualities compared with the recurrent parent. Moreover, the improved hybrids derived from the NILs exhibited better agronomic performance and rice quality compared with the controls under natural field conditions.

**Conclusions:**

This study demonstrates that it is essential to stack *Bph14* and *Bph15* into both the maternal and paternal parents for developing BPH-resistant hybrid rice varieties. The SNP array with abundant DNA markers is an efficient tool for analyzing the RPG recovery of progenies and can be used to monitor the donor segments in NILs, thus being extremely important for rice molecular breeding.

## Background

Rice (*Oryza Sativa* L.) is one of the most important food sources for more than half of the world population. It is predicted that an additional 116 million tons of rice will be needed by 2035 to feed the growing population (Seck et al. 2012). However, the brown planthopper (BPH, *Nilaparvata lugens* Stål), which sucks the phloem sap of the rice leaf sheath and transmits viral diseases such as rice grassy stunt virus (RGSV), rice ragged stunt virus (RRSV) and rice wilted stunt virus (RWSV), often leads to severe yield losses in the agricultural industry (Fujita et al. 2013).

More than 90 % of the world’s rice is grown in Asian countries. China is the largest rice-producing country in the world, although the planting area is less than that in India. The total rice production in 2015 was about 208 million tons in China, which accounted for more than 20 % of the world’s total rice yield. Widely grown hybrid rice is one of the main factors contributing to the increase in rice yield in China since the 1970s. The IRRI estimates that planthoppers cause annual yield losses of 1 to 2 million tons of paddy rice in China (Fujita et al. 2013). The total planting area of rice infested by BPH in China exceeded 25 million hectares between 2005 and 2007 (Qiu et al. 2012). Rapid expansion of the planting area of hybrid rice is probably one of the main causes of the BPH crisis because most of the hybrid rice varieties released in China are susceptible to BPH (Hu et al. 2012). The area planted to hybrid rice has already increased by over 50 % in China, and the planted area of hybrid rice in India and other Asian countries such as Vietnam, Myanmar and Indonesia, has also increased in recent years (Horgan and Crisol 2013). Therefore, it is a very urgent task to develop hybrid rice cultivars with BPH resistance as soon as possible.

Fujita et al. (2013) reviewed the progress in the identification of BPH resistance genes. To date, 29 major BPH resistance genes/loci have been identified from cultivated, landrace and wild species of *Oryza* (Fujita et al. 2013; Wang et al. 2015). These are located on 8 of the 12 chromosomes in rice, whereas no BPH genes have been identified on chromosomes 1, 5, 7 and 8. Multiple BPH-resistance loci are closely positioned or overlap on the same chromosome regions, and four gene clusters have been designated as A, B, C and D (Fujita et al. 2013). The continuing validation of BPH-resistance loci offers possibilities for rice molecular breeding.

Compared with traditional chemical control, a host plant resistance breeding strategy is a more effective and environmentally friendly method to control BPH damage (Jairin et al. 2010; Sun et al. 2005). Molecular marker-assisted selection (MAS) is a highly efficient approach for plant-breeding scientists to select target genes both rapidly and precisely (Tanksley et al. 1989). It has been certified that pyramiding of resistance genes can provide stronger and more durable resistance for susceptible rice cultivars in contrast to single gene introgression. Myint et al. (2012) found that the BPH resistance level of *Bph25* + *Bph26*-NILs was significantly higher than *Bph25*-NILs or *Bph26*-NILs. Qiu et al. (2012) found that the *indica* rice 9311 and *japonica* rice Nipponbare lines with pyramided *Bph6* and *Bph12* had obviously less damage compared to monogenic lines.

*Bph14* and *Bph15*, separately located on chromosome 3 and 4 in B5 (a breeding line derived from *O. officinalis*), are two widely adopted genes in the BPH-resistance breeding practice (Huang et al. 2001; Li et al. 2006; Xia et al. 2010; Zhu et al. 2013a; Zhu et al. 2013b; Hu et al. 2012; Hu et al. 2015; Cai et al. 2015). *Bph14* was the first cloned BPH resistance gene and encodes a coiled-coil, nucleotide-binding and leucine-rich repeat (CC-NB-LRR) protein (Du et al. 2009). *Bph15* was previously mapped to a 47-kb region, but the latest research showed that the region containing *Bph15* was nearly 580 kb between DNA marker g12140-2 and marker T12 in B5, which originated from the C genome of *O. officinalis* (Yang et al. 2004; Lv et al. 2014). A previous study found that the BPH-resistance effect of *Bph15* was higher than that of *Bph14*. Moreover, both *Bph14* and *Bph15* exhibit partial dominance and have a pronounced dosage effect on the resistance to BPH in hybrids (Li et al. 2011; Hu et al. 2012).

Huahui938 is an elite rice restorer line of CMS selected by our laboratory in previous years. We developed several hybrids with high yield, good quality and resistance to blast using Huahui938 as a male parent. However, Huahui938 and its derived hybrids are susceptible to BPH. In the present study, we further improved the BPH-resistance of Huahui938 and its derived hybrids by pyramiding *Bph14* and *Bph15* using marker-assisted backcross selection (MAB) and whole-genome SNP array analysis, following the effective evaluation of pyramided genes conferring resistance to BPH in the improved NILs and the corresponding hybrids.

## Results

### Development of NILs with *Bph14* and *Bph15* Genes Using MAB

**Fig. 1:**
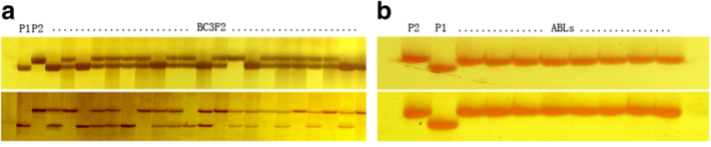
PCR analysis of BC_3_F_2_ plants from the cross between Huahui938 and B5 (**a**) and resistance gene confirmation of ABLs in BC_3_F_3_ (**b**). **a** Upper panel: PCR analysis of *Bph14* with marker 76-2; Lower panel: PCR analysis of *Bph15* with marker MS5; **b** Upper panel: *Bph14* gene confirmation with marker 76-2; Lower panel: *Bph15* gene confirmation with marker InD4. P_1_: Huahui938; P_2_: B5; ABLs: advanced backcrossed breeding lines

F_1_ progenies were obtained from the cross between Huahui938 (recurrent parent) and B5 (donor parent of *Bph14 and Bph15*). Based on PCR analysis, true F_1_ plants containing *Bph14* and *Bph15* were selected and backcrossed with Huahui938 to produce BC_1_F_1_. Among 30 BC_1_F_1_ plants, five containing heterozygous *Bph14* and *Bph15* loci were used for further backcrossing to produce BC_2_F_1_. Among 100 BC_2_F_1_ plants, two individuals containing *Bph14* and *Bph15* and with a phenotype similar to Huahui938 were used for subsequent backcrossing. Among 60 BC_3_F_1_ plants, 19 were identified as containing *Bph14* and *Bph15,* and two plants (designed as HB13002-5 and HB13002-50) with identical agronomic performance to Huahui938 were self-crossed to produce BC_3_F_2_ seeds. Then, in two BC_3_F_2_ populations of 100 plants, eight plants containing homozygous *Bph14* and *Bph15* were selected to produce BC_3_F_3_ (Fig. 1a). Next, eight BC_3_F_3_ family lines were obtained and further confirmed by PCR analysis of the target loci, which suggested that both *Bph14* and *Bph15* were homozygous in all family lines (Fig. 1b). Finally, three lines with no phenotype segregation of agronomic performance, including number of days to heading, plant height, tiller number, plant type and panicle type, among individuals of the population were designed as HB13002-5-30-10, HB13002-5-31-24 and HB13002-50-9-7.

**Fig. 2:**
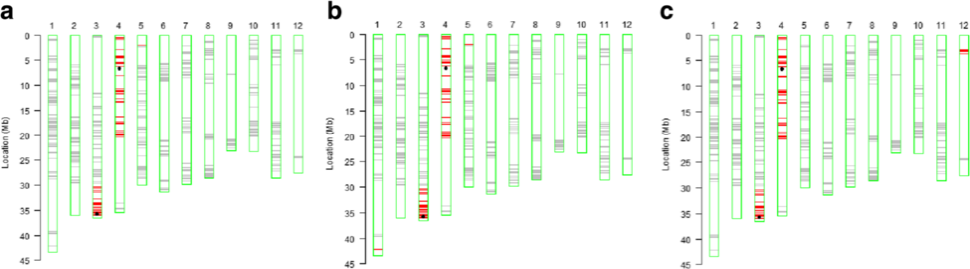
Genetic background assay of the three selected NILs detected using the RICE6K array. **a** HB13002-5-30-10, **b** HB13002-5-31-24 and **c** HB13002-50-9-7. The *black dots* indicate the positions of two target genes, *Bph14* on chromosome 3 and *Bph15* on chromosome 4. The *red lines* indicate the SNP loci with homozygous genotypes where genomic fragments of the donor parent B5 were introgressed, the *gray lines* indicate the SNP loci with the same genotypes as the recurrent parent Huahui938, and the *white boxes* indicate non-polymorphism fragments between recurrent and donor parents

The genetic backgrounds of the three selected near-isogenic lines (NILs) were assayed using the whole-genome SNP array RICE6K. A total of 550 SNP markers showed polymorphism between Huahui938 and B5. The recurrent parent genome (RPG) recoveries of HB13002-5-30-10, HB13002-5-31-24 and HB13002-50-9-7 were 87.88, 87.70 and 86.62 %, respectively. The haplotype maps also implied that the segments containing target loci were successfully transferred from the donor parent B5 into the selected NILs, and no heterozygous loci remained at the whole-genome scale of the three improved lines (Fig. 2). These results suggested that positive selection of target genes and continuous phenotypic selection can be used to achieve the genetic background of the recurrent parent with a recovery rate higher than 80 % and can also be used to rapidly establish the whole-genome homozygosity of the selected lines.

### Resistance Reaction of the Selected NILs and Hybrids in Response to BPH

**Fig. 3:**
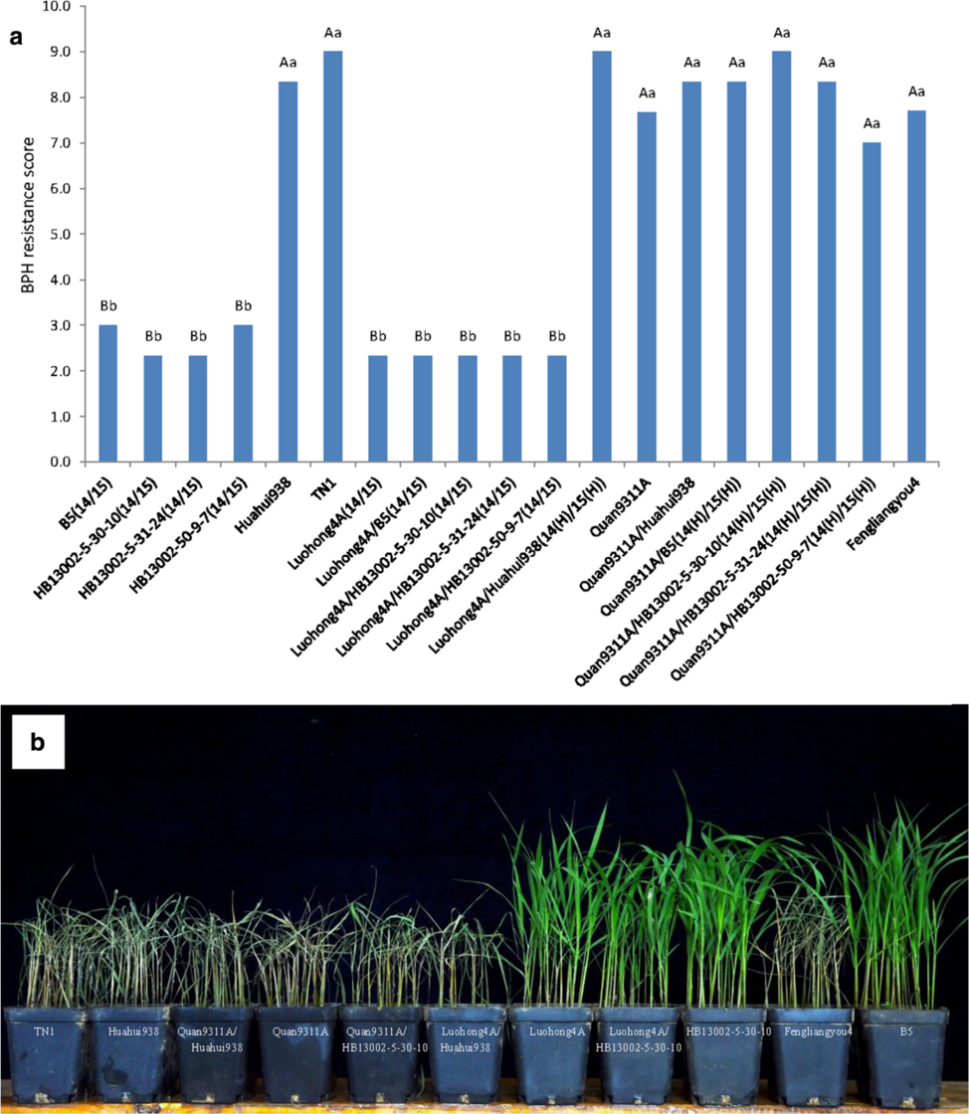
The evaluation results of BPH resistance of the selected NILs and their derived hybrids seven days after infestation. **a** The BPH resistance scores of the selected NILs, their derived hybrids, parents and controls. Lower scores indicate higher resistance to BPH. Data are the means of three replicates; A and B indicate ranking according to Duncan’s test at *P* < 0.01; a and b indicate ranking according to Duncan’s test at *P* < 0.05. 14/15 in parentheses: homozygous state of both *Bph14* and *Bph15*, 14(H)/15(H): heterozygous state of both *Bph14* and *Bph15*. **b** The performance (showing the damage degree) of HB13002-5-30-10 (a selected NIL) and its two derived hybrids, Huahui938 (recurrent parent) and its two derived hybrids, B5 (donor parent), Luohong4A, TN1 (susceptible control) and Fengliangyou4 (a popular hybrid)

The resistance levels to BPH of the selected NILs and their derived hybrids, along with the controls, were evaluated at the seedling stage. The evaluation results showed that B5 and the CMS line Luohong4A with two homozygous resistance genes were resistant against BPH, with scores of 3.0 and 2.3, respectively. The three selected NILs, HB13002-5-30-10, HB13002-5-31-24 and HB13002-50-9-7, were also resistant against BPH, similar to the resistance controls, with scores of 2.3, 2.3 and 3.0, respectively. Huahui938, TN1, Quan9311A, Fengliangyou4 and Quan9311A/Huahui938, without resistance genes, were susceptible or highly susceptible to BPH, and their scores ranged from 7.7 to 9.0. Interestingly, the hybrids with homozygous *Bph14* and *Bph15* genes, i.e., Luohong4A/B5, Luohong4A/HB13002-5-30-10, Luohong4A/HB13002-5-31-24 and Luohong4A/HB13002-50-9-7, were resistant against BPH, and their BPH resistance scores were 2.3-3.0. However, the hybrids containing heterozygous *Bph14* and *Bph15* genes, i.e., Luohong4A/Huahui938, Quan9311A/B5, Quan9311A/HB13002-5-30-10, Quan9311A/HB13002-5-31-24 and Quan9311A/HB13002-50-9-7, were susceptible or highly susceptible to BPH, similar to the materials without BPH-resistant genes (Fig. 3a and b). Taken together, these results indicate that the protection of hybrid rice from damage caused by BPH requires the simultaneous genetic improvement of maternal and paternal parents with the use of *Bph14* and *Bph15*.

### The Performance of Agronomic Traits of the NILs and Their Hybrids

To test whether the other phenotypic traits of the selected NILs and their derived hybrids were identical to those of the recurrent parent and its derived hybrids under normal conditions, we examined eight agronomic traits and six rice quality traits of the selected NILs and hybrids planted at Wuhan without BPH damage (Table 1). Traits such as panicle length (PL), panicle number per plant (PN), number of grains per panicle (NGP), fertility of the spikelet (FER), 1000-grain weight (GW), grain yield per plant (GY), chalkiness degree (CD), amylose content (AC) and alkali spreading value (ASV) of the three selected NILs showed no significant difference compared with Huahui938. However, the number of days to heading (DTH) of HB13002-50-9-7 was 3 days longer than that of Huahui938, the PN of HB13002-50-9-7 was 2.8 lower than Huahui938 and the plant height (PH) of HB13002-30-10 was approximately 5.3 cm higher than that of Huahui938. Moreover, the head rice rate (HR) of the three selected NILs was higher than that of Huahui938, which is a desirable quality trait for rice improvement.

**Table 1:** The agronomic and rice quality traits of the three selected NILs and their derived hybrids

Entire	Agronomic traits								Traits of grain qualities					
	DTH (d)	PH (cm)	PL (cm)	PN	NGP	FER (%)	GW (g)	GY (g)	HR (%)	L/W	CK (%)	CD (%)	AC (%)	ASV
Huahui 938	92.0^b^	117.5^c^	27.1^b^	14.4^b^	137.2^a^	77.66^a^	24.6^b^	29.2 ^a^	53.9^c^	2.87^b^	11.4^bc^	4.0^b^	10.2^b^	6.0^a^
HB13002-5-30-10	91.7^b^	122.8^a^	28.1^ab^	13.3^bc^	149.3^a^	81.07^a^	25.6^b^	30.0^a^	57.8^b^	3.03^a^	13.9^b^	4.2^b^	10.6^ab^	6.0^a^
HB13002-5-31-24	92.0^b^	118.6^bc^	27.3^ab^	13.4^bc^	137.8^a^	77.39^a^	24.9^b^	29.8^a^	59.0^ab^	2.90^b^	11.0^bc^	3.1^b^	10.2^b^	5.9^a^
HB13002-50-9-7	95.0^a^	120.3^bc^	28.3^ab^	11.6^d^	145.2^a^	82.04^a^	25.1^b^	28.3^a^	58.1^b^	2.90^b^	8.7^c^	3.5^b^	10.7^ab^	6.0^a^
B5	86.7^c^	113.8^d^	28.8^a^	16.5^a^	115.7^b^	81.05^a^	27.6^a^	29.4^a^	61.1^a^	2.77^c^	34.0^a^	10.5^a^	11.1^a^	2.0^b^
Luohong4A/Huahui938	84.0^b^	105.3^bc^	25.1^ab^	11.1^b^	180.8^bc^	79.93^a^	25.2^bc^	37.2^a^	61.4^b^	2.87^a^	53.7^a^	21.2^a^	20.9^a^	3.4^bc^
Luohong4A/HB13002-5-30-10	84.0^b^	106.4^b^	25.3^ab^	11.4^b^	171.7^c^	75.93^a^	25.6^b^	33.8^b^	65.1^a^	2.90^a^	31.5^c^	11.0^c^	19.2^b^	3.8^b^
Luohong4A/HB13002-5-31-24	83.3^b^	103.0^c^	25.3^ab^	11.5^b^	176.2^bc^	70.77^bc^	25.6^b^	33.7^b^	65.1^a^	2.90^a^	37.0^bc^	12.3^bc^	19.3^b^	3.1^bc^
Luohong4A/HB13002-50-9-7	84.7^b^	106.5^b^	25.5^a^	11.3^b^	186.6^b^	74.93^ab^	24.7^c^	35.3^ab^	65.8^a^	2.90^a^	29.4^c^	11.2^bc^	20.9^a^	3.7^bc^
Luohong4A/B5	80.1^c^	102.9^c^	24.7^b^	13.0^a^	154.0^d^	63.4^c^	26.9^a^	27.0^c^	65.6^a^	2.87^a^	45.8^ab^	13.8^b^	19.1^b^	2.7^c^
Fengliangyou4(CK)	88.0^a^	117.3^a^	24.9^ab^	9.6^c^	203.7^a^	81.62^a^	27.46^a^	35.2^b^	64.5^a^	2.77^b^	29.2^c^	8.3^d^	14.9^c^	5.9^a^
Quan9311A/Huahui938	88.7^a^	115.2^a^	27.3^b^	12.0^a^	181.1^bc^	82.89 ^ab^	27.5^b^	38.6^ab^	64.3^a^	2.77^a^	18.0^c^	6.1^b^	13.2^b^	5.8^a^
Quan9311A/HB13002-5-30-10	88.7^a^	115.0^a^	27.9^ab^	11.6^a^	179.5^bc^	86.11^a^	28.4^a^	39.7^ab^	64.6^a^	2.73^a^	17.2^c^	6.0^bc^	13.3^b^	6.2^a^
Quan9311A/HB13002-5-31-24	88.7^a^	114.9^a^	28.2^ab^	10.7^ab^	188.9^ab^	84.61^ab^	27.3^b^	40.0^ab^	65.1^a^	2.80^a^	16.0^c^	6.0^c^	12.5^c^	6.1^a^
Quan9311A/HB13002-50-9-7	88.7^a^	114.9^a^	27.9^ab^	12.1^a^	182.1^bc^	84.11^ab^	27.9^ab^	43.3^a^	65.4^a^	2.80^a^	22.9^b^	6.2^a^	13.2^b^	6.0^a^
Quan9311A/B5	87.7^b^	110.5^b^	28.5^a^	11.2^ab^	172.3^c^	48.39^c^	28.2^a^	20.8^c^	64.1^a^	2.67^b^	7.5^d^	5.9^d^	11.7^d^	4.8^b^
Fengliangyou4(CK)	88.0^b^	117.3^a^	24.9^c^	9.6^b^	203.7^a^	81.62^b^	27.5^b^	35.2^b^	64.5^a^	2.77^a^	29.2^a^	6.1^bc^	14.9^a^	5.9^a^

*DTH* Number of days to heading, *PH* plant height, *PL* panicle length, *PN* panicle number per plant, *NGP* number of grains per panicle, *FER* fertility of the spikelet (%), *GW* weight of 1000 grains, *GY* grains yield per plant, *HR* head rice rate (%), *L/W* ratio of length to width, *CK* chalky grain rate, *CD* chalkiness degree, *AC* amylose content, *ASV* alkali spreading value. ^a, b, c, d^ Means followed by the same letter are not significant at the 5 % significance level by the least significant difference test (LSD = 0.05)

The evaluation results of the agronomic and rice quality traits of hybrids derived from the selected NILs showed that most of traits were identical to those of the hybrids derived from the recurrent parent, Huahui938 (Table 1). Among them, the GY of improved hybrid Luohong4A/HB13002-9-7 was not significantly different from that of the control Luohong4A/Huahui938. In addition, all three improved hybrids had better rice quality with a higher HR and significantly lower CK and CD compared with Luohong4A/Huahui938. Furthermore, most of the agronomic traits of the three improved Quan9311A/NILs were identical to the control Quan9311A/Huahui938 except that Quan9311A/ HB13002-30-10 had a higher GW. Additionally, all three hybrids derived from Quan9311A had a better rice quality with a significantly lower chalky grain rate (CK). Compared to Fengliangyou4, a popular and outstanding hybrid variety in agricultural production, the hybrid Luohong4A/HB13002-9-7 showed similar GY but shorter DTH and lower PH. Meanwhile, the hybrid Quan9311A/HB13002-9-7 had a significantly higher GY and lower CK compared with Fengliangyou4 (Table 1). The BPH-resistant NILs and hybrids, which were identical to the recurrent parent and its derived hybrids with respect to the agronomic and rice quality traits, are more strongly desired.

## Discussions

BPH has been one of the important factors constraining rice yield. As the area of cultivated hybrid rice varieties gradually expands around the world, their resistance to BPH is attracting increasing attention by rice researchers (Hasan et al. 2015). Numerous BPH resistance genes have been introduced and pyramided into elite rice varieties, which has resulted in strongly improved capacity of rice to endure damage caused by BPH (Myint et al. 2012; Qiu et al. 2012; Wan et al. 2013; Suh et al. 2011). With the adaptation of BPH to resistant varieties and the evolution of a new biotype of BPH (Fujita et al. 2013), different BPH resistance genes still need to be pyramided into multiple elite hybrid rice varieties to overcome these problems. In the present study, through molecular-marker assisted backcross (MAB) breeding, we successfully transferred two BPH-resistance genes, *Bph14* and *Bph15*, into an elite restorer line Huahui938 to obtain three improved lines, which showed enhanced resistance to BPH compared with the controls. When these NILs were crossed with the CMS line with the same BPH resistance genes, the hybrids (homozygous genotype with resistance loci) were resistant against BPH. Some derived hybrids had higher yield and more desirable rice qualities and agronomic performance compared with the controls. Therefore, it is possible to develop hybrid rice with high BPH resistance and excellent agronomic performance in the future and to solve the problem of hybrid rice being susceptible to BPH (Horgan and Crisol 2013).

The effect of BPH resistance genes would be influenced by various genetic backgrounds (Qiu et al. 2012; Jairin et al. 2010). Some previous studies demonstrated that hybrids with heterozygous *Bph14* and *Bph15* showed moderate resistance to BPH (Hu et al. 2012; Hu et al. 2013). However, our results indicated that the heterozygote of these two genes was susceptible to BPH in some hybrid backgrounds at the seedling stage. In fact, BPH resistance genes with a partial dominant effect would show moderate resistance or would be susceptible to BPH in a heterozygous state under different genetic backgrounds (Liu et al. 2016). Therefore, it is essential to simultaneously stack *Bph14* and *Bph15* into male and female parents to guarantee hybrids with certain and durable resistance to BPH.

With respect to rice genetic improvement, developing NILs is the optimal approach to obtain modified lines with desired traits. Molecular marker-assisted selection (MAS) is a very powerful tool to accelerate the progress of developing NILs with pyramided resistance genes and to maintain the desirable characteristics of the recurrent parent. Numerous NILs have been developed with enhanced disease resistance, such as blast resistance, sheath blight resistance, and bacterial leaf blight resistance, under different genetic backgrounds (Miah et al. 2015; Khanna et al. 2015; Chen et al. 2014; Suh et al. 2013; Balachiranjeevi et al. 2015).

In previous studies in which NILs were developed, hundreds of RFLP or SSR markers were usually used for recovering and detecting the genetic background. It is time-consuming and low-efficiency work to identify polymorphism markers between the donor and recipient parent (Chen et al. 2000; Suh et al. 2011; Ahmed et al. 2016). However, it is easy to identify sufficient polymorphism SNP markers for background analysis due to the high-density and extensive marker positioning in an SNP array. In a comparative study estimating the similarity between the recurrent parent and the developed NILs, Khanna et al. (2015) found that the percent similarity was overestimated by SSR markers, and the background analysis using an SNP array was almost 300 times more cost effective and provided a more precise estimation due to higher resolution.

In this study, the genetic background of the improved NILs was analyzed using the SNP array RICE6K, which contains 5,102 SNP and InDel markers. We detected 550 SNP markers showing polymorphisms between Huahui938 and B5 in an efficient amount of time. Jiang et al. (2015) examined the genetic background recovery of developed lines using the same RICE6K array and obtained accurate results. Mi et al. (2016) obtained a modified 9311 line carrying stacked genes *f5-n* and *S5-n* with an RPG recovery rate higher than 99 % using RICE6K, and a higher density SNP array in the BC_5_ generation and the agronomic performance of those NILs did not differ from the recurrent parent 9311. Thus, as the cost of genotyping using an SNP array decreases, it will be possible to use this powerful tool to develop NILs in all breeding schemes in the near future.

In the present study, the hybrid combination Luohong4A/HB13002-50-9-7 exhibited BPH resistance, high yield and good rice quality similar to that of Fengliangyou4. We affirm that this hybrid can be used for rice production in the BPH epidemic region in southern China. The yield of Quan9311A/HB13002-50-9-7 was the highest among the tested combinations even though it showed no resistance against BPH at the seedling stage. We believe this can be used as a high yield hybrid for production in areas where BPH does not occur.

## Conclusions

In this study, we successfully transferred both *Bph14* and *Bph15* into the CMS rice restorer line, and three BPH-resistance NILs were obtained. The results showed that when both male and female parents contained the same BPH resistance genes, the hybrids were resistant against BPH. The combination Luohong4A/HB13002-50-9-7 obtained in this study had high yield, BPH resistance, superior rice quality and desirable agronomic performance, showing a remarkable commercial potential for hybrid rice production in BPH epidemic areas.

## Methods

### Rice Materials and Breeding Scheme

**Fig. 4:**
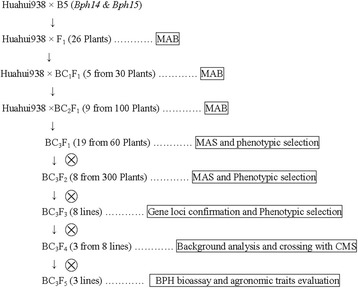
Procedure for the development of NILs with homozygous *Bph14* and *Bph15* loci in the genetic background of Huahui938

Huahui938, an elite *indica* CMS rice restorer line with good combined abilities of yield, good rice quality and high blast resistance but susceptible to BPH, was used as the recurrent parent. B5, a highly resistant line whose resistance genes *Bph14* and *Bph15* were obtained from wild rice (*Oryza officinalis*), was used as the donor parent (Huang et al. 2001). Taichung Native 1 (TN1), a line highly susceptible to BPH, was used as the negative control to evaluate BPH resistance. Two CMS lines, Quan9311A and Luohong4A, were used as the maternal parents to produce the hybrid rice. Quan9311A was developed by the Quanyin Seed Company, Hefei, China, and has good combined abilities of yield and high rice quality but is susceptible to BPH. Luohong4A, which was developed by Wuhan University, Wuhan, China, harbors homozygous *Bph14* and *Bph15* loci and is resistant to BPH (Zhu et al. 2013). In addition, Fengliangyou4, which was developed by the Fengle Seed Company, Anhui province, China, and is an existing check variety for the registration of new rice varieties in China, was used as the control for BPH resistance and agronomic performance evaluation of hybrid rice in this study. The overall breeding scheme consisted of a recurrent backcrossing procedure including one crossing, three generations of backcrossing and four generations of self-fertilization, combined with MAS in each generation (Fig. 4). A cross was made between the recurrent parent Huahui938 and the donor parent B5. In backcrossing generations from BC_1_F_1_ to BC_3_F_1_, selected individuals with a phenotype similar to Huahui938 and heterozygous *Bph14* and *Bph15* loci were backcrossed to Huahui938. Three near-isogenic lines (NILs) bearing both homozygous *Bph14* and *Bph15* loci were generated from the BC_3_F_2_ segregation population. Finally, the RICE6K array was used to verify the background of the improved lines.

### DNA Extraction, PCR, Markers and Genotyping

Total genomic DNA was extracted from fresh rice leaves using the modified CTAB method following Dellaporta et al. (1983). PCR reactions were carried out as described by Jiang et al. (2015). PCR reactions were performed using a MyCycler™ thermal cycler (BIO-RAD USA) with 20 μl reaction mixture that contained 20 ng genomic DNA, 10 mM Tris-HCl (pH 9.0), 50 mM KCl, 2.5 mM MgCl_2_, 2 mM dNTPs, 10 μM each of the forward and reverse primers, and 1 unit of Taq DNA polymerase. The PCR amplification program consisted of one cycle of denaturation at 95 °C for 5 min, followed by 35 cycles at 95 °C for 30 s, 56 °C for 30 s, 72 °C for 45 s, with a final extension at 72 °C for 7 min. The InDel marker 76-2 (primer sequences F: 5′-GCACATACAGAAATGGTGAA-3′, R: 5′-GGCAAGGGACATGTAGTAAC-3′) linked to *Bph14* was used to select positive individual plants with respect to the *Bph14* locus in each generation (Du et al. 2009). The SSR marker MS5 (primer sequences F: 5′- TTGTGGGTCCTCATCTCCTC-3′, R: 5′-TGACAACTTGTGCAAGATCAAA-3′) linked to *Bph15* (Yang et al. 2004) and the InDel marker InD4 (primer sequences F: 5′-AGAATGCTAAAGATGACTGAA-3′, R: 5′-AACGGTATTGTTCTTGTCTAA-3′), which was more tightly linked to *Bph15* (Lv et al. 2014), were used to select individual positive plants in each generation. The PCR products were analyzed using 4 % denaturing polyacrylamide gel electrophoresis. In addition, the whole-genome single nucleotide polymorphism (SNP) array RICE6K was used to analyze the genetic background similarity of the selected NILs with respect to the recurrent parent Huahui938. This SNP array was developed based on Infinium technology, which contains 5102 SNP and insertion-deletion (InDel) markers evenly distributed on the 12 chromosomes of rice with an average density of 12 SNPs per 1 Mb (Yu et al. 2014). For each improved line, total DNA was extracted from 20 plants leaves. Genetic background similarity analysis was performed at the Life Science and Technology Center, China National Seed Group Co., LTD (Wuhan, China), according to Infinium HD Assay Ultra Protocol (http://www.illumina.com/).

### BPH Bioassay for the Selected NILs and Their Hybrids

The BPH resistance evaluations of the selected NILs and their hybrids were conducted in a net house. Huahui938, Quan9311A, Fengliangyou4 and TN1 were used as susceptible controls, while Luohong4A and the donor parent B5 were used as the BPH resistant controls. The BPH samples used in the test were collected from the field and were continuously reared on TN1 in the laboratory at Huazhong Agricultural University (HZAU), Wuhan, China. The bioassay was performed using a modified seedling bulk test following the SSST (Velusamy et al. 1986; Horgan 2009). Thirty pre-germinated seeds of each entry, including the improved lines and controls with three replications, were evenly sown in 7 × 7 × 8 cm plastic plates. All entries were randomly arranged in a large pool. When the seedlings reached the second leaf stage, they were thinned to 20 plants per plate. At the third-leaf stage, the seedlings were infested with 2nd-3rd-instar BPH nymphs at a rate of 8–10 insects per seedling, and the water was maintained at a depth of approximately 0.5 cm above the root until the evaluation was completed. All seedlings were patted once, 24 h after the infestation, to evenly redistribute the nymphs. When all TN1 plants died, the other plants were examined and the following scores were given: 0 (no damage), 1 (very slight damage), 3 (the first and second leaf of most plants were yellowing), 5 (yellowing, nearly half of the plants had wilted or were dead), 7 (more than half of the plants were dead and the rest were seriously dwarfed) and 9 (all plants were dead) according to the modified criteria based on the Standard Evaluation System for Rice (IRRI 2002). An average resistance score of 0.1–1.9 was designated as highly resistant (HR), 2.0–3.9 as resistant (R), 4.0–5.9 as moderately resistant (MR), 6.0–7.9 as susceptible (S), and 8.0–9.0 as highly susceptible (HS) (IRRI 2002).

### Evaluation of Agronomic and Grain Quality Traits

To evaluate the agronomic and rice quality traits, Huahui938, B5, three improved NILs and their derived hybrids from Quan9311A and Luohong4A were planted in a randomized complete block design with three replications at Wuhan, China, in the summer of 2015 under natural field conditions. Each plot consisted of three rows with 10 plants per row at a planting density of 16 cm between plants within a row and 20 cm between rows. The agronomic and rice quality traits were measured according to the standard evaluation system for rice (IRRI 2002). Five mature plants in the middle row were harvested for measurements. The agronomic traits evaluated included number of days to heading (DTH), plant height (PH), panicle length (PL), panicle number (PN), number of grains per panicle (NGP), fertility of the spikelet (FER), grain yield (GY), and 1000-grain weight (GW). The harvested seeds were stored at room temperature for three months at 14 % moisture content. The following grain quality traits were tested: head rice rate (HR), ratio of grain length to width (L/W), chalky grain rate (CK), chalkiness degree (CD), amylose content (AC) and alkali spreading value (ASV).

### Data analysis

The background recovery percentage (BRP) was calculated using the following formula: BRP (%) = (R + 1/2H) × 100/P. In this formula, R, H, and P indicate the number of SNP markers homozygous for Huahui938, the number of markers that remained heterozygous, and the total number of polymorphic markers between Huahui938 and B5, respectively. One-way ANVOA and least significant difference (LSD) tests were performed using the SAS statistical software package (version 9.0; SAS Institute, Cary, NC).

## Abbreviations

AC: Amylose content
ASV: Alkali spreading value
BPH: Brown planthopper
BRP: Background recovery percentage
CD: Chalkiness degree
CK: Chalky grain rate
CMS: Cytoplasmic male sterility
CTAB: Hexadecyl trimethyl ammonium bromide
DTH: Number of days to heading
FER: Fertility of the spikelet
GW: Grain weight
GY: Grain yield
HR: Head rice rate
Indel: Insertion-deletion
IRRI: International Rice Research Institute
L/W: Ratio of grain length to width
LSD: Least significant difference tests
MAB: Molecular marker-assisted backcross
MAS: Marker-assisted selection
NGP: Number of grains per panicle
NILs: Near-isogenic lines
PCR: Polymerase chain reaction
PH: Plant height
PL: Panicle length
PN: Panicle number
RGSV: Rice grassy stunt virus
RPG: Recurrent parent genome
RRSV: Rice ragged stunt virus
RWSV: Rice wilted stunt virus
SES: Standard evaluation system
SNP: Single nucleotide polymorphism
SSR: Simple sequence repeat
SSST: Standard Seedbox Screening Test
TGMS: Thermo-sensitive genetic male-sterile lines
